# An aerogel-based light-driven knudsen pump approaching complete passive capturing of airborne particles in wide size ranges

**DOI:** 10.1016/j.fmre.2025.04.006

**Published:** 2025-04-18

**Authors:** Yi-Bo Zhao, Tingting Wu, Céline Heimhofer, Natascha Heppeler, Min Gao, Tianyu Cen, Fabian Weyand, Christian Ludwig, Shanyu Zhao, Jing Wang

**Affiliations:** a Institute of Environmental Engineering, ETH Zürich, Zürich 8093, Switzerland; b Laboratory for Nano Particles, Empa, Swiss Federal Laboratories for Materials Science and Technology, Dübendorf 8600, Switzerland; c Department of Mechanical Engineering, University of Minnesota, Minneapolis 55455, MN, USA; d Laboratory for Building Energy Materials and Components, Empa, Swiss Federal Laboratories for Materials Science and Technology, Dübendorf 8600, Switzerland; e Institute of Sustainability for Chemicals, Energy and Environment (ISCE^2^), Agency for Science, Technology and Research (A*STAR), Jurong Island 627833, Singapore; f Center for Energy and Environmental Sciences, Paul Scherrer Institut (PSI), Villigen PSI 5232, Switzerland; g Environmental Engineering Institute (IIE, GR-LUD), School of Architecture, Civil and Environmental Engineering (ENAC), École Polytechnique Fédérale de Lausanne (EPFL), Lausanne 1015, Switzerland; h Department of Chemistry and Biology, Federal Institute of Metrology (METAS), Bern-Wabern 3003, Switzerland

**Keywords:** Polyimide aerogel, Tunable pores, Passive sampler, Light-driven, Aerosol particles, Water-soluble metals

## Abstract

Aerosol sampling stands as an essential step in air quality monitoring and characterization. Active sampling provides accurate aerosol determination, while passive sampling offers cost-effective and power supply-free design. However, passive sampling is subject to sampling conditions and brings about uncertainties concerning particle sizes and flow rates, and collection efficiencies, which requires calibration assisted by active samplings. In this work, we presented a polyimide (PI) aerogel-based passive sampler operating in a flow-through and active-sampling mode without artificial power supply, generating a gas flow rate in the range of mL min^-1^ cm^-2^ (volume flow rate/surface area of PI aerogel) or cm min^-1^, induced by light-driven thermal transpiration effects. It demonstrated a remarkably high collection efficiency of airborne particles approaching 100% in the particle range of 10 nm–10 µm, enabling the calibration-free sampling of airborne particles without requiring an additional power supply. We explored the effects of light intensity and pore size on the sampling performance, establishing a relationship between temperature gradient and flow rate. An analysis of the distribution and penetration of NaCl particles and real-world particles was conducted at the micrometer level, which exhibits a main penetration depth of < 100 µm. With further modifications and scaling-up of the PI aerogel passive sampler for ambient applications, it holds great potential to function independently for sampling and analysis of water-soluble airborne compounds (e.g. Mg and Fe) and promote the extensive and cost-effective air quality monitoring and control.

## Graphical abstract

**Figure:**
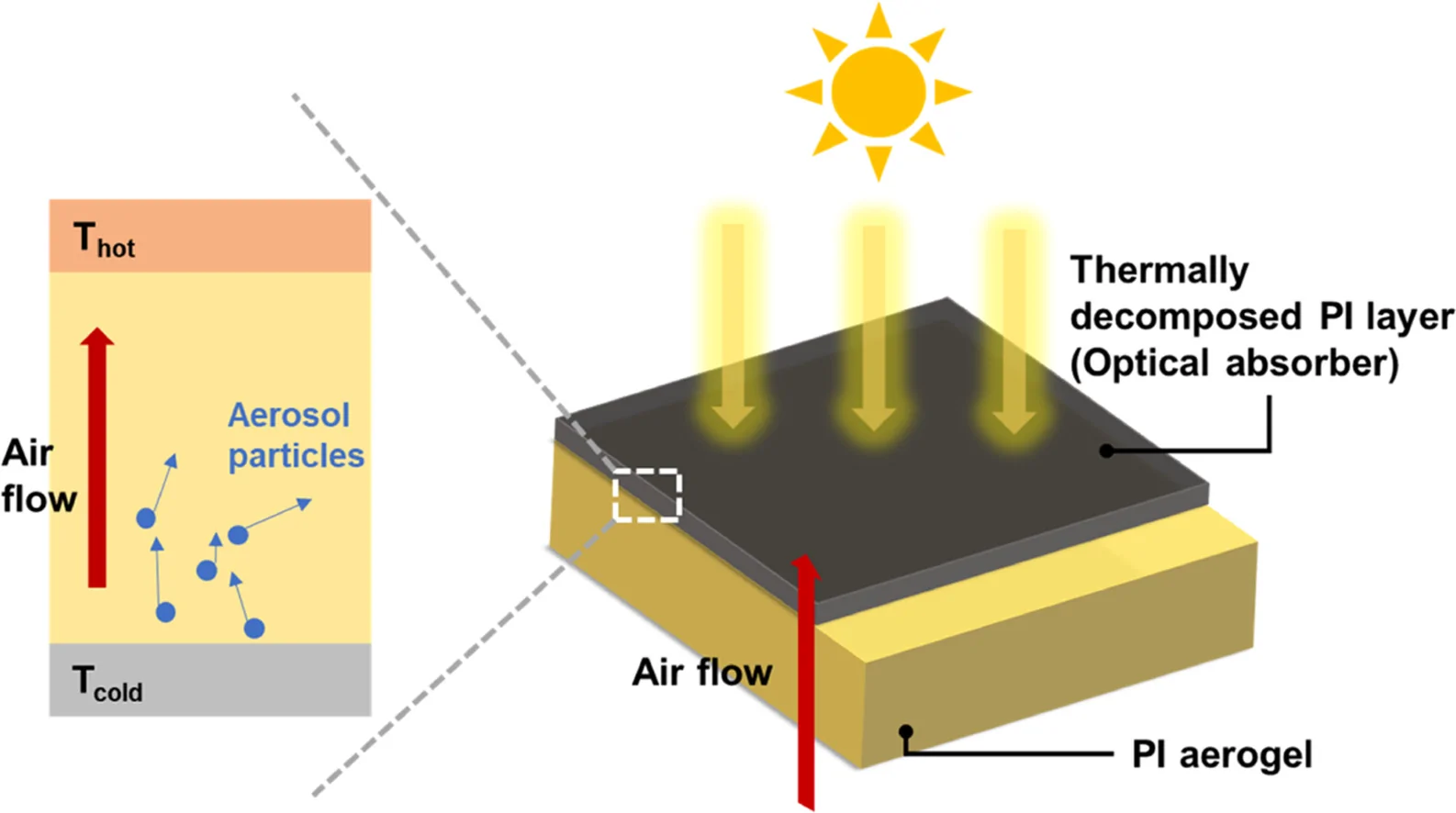

## 1 Introduction

The high level of particulate matter is one of the most significant global health risks, increasing chronic diseases (e.g., cardiovascular and pulmonary diseases) and premature deaths [1,2]. Aerosol sampling is an essential step for air quality monitoring and control, which consists of active and passive aerosol sampling. Compared to active aerosol sampling, passive aerosol sampling enables the characterization of time-integrated concentrations, distributions, and spatiotemporal trends with minimal maintenance in a power-free and cost-effective manner [3,4]. Passive aerosol sampling can provide the time-weighted average concentrations for estimating long-term exposures of low-concentration pollutants and extensive monitoring on regional, continental, and global scales [5].

Passive air samplers have been widely used in the collection and study of persistent organic pollutants [[6], [7], [8]], airborne particles [[9], [10], [11]], bioaerosols [12,13], microplastics [14], and metals [5,15] in the atmosphere. Nonetheless, passive aerosol samplers without quantitative sampling flowrates rely on sampling mechanisms such as diffusion, gravitational force, and electrostatic forces, and passive air sampling rate could be subject to a number of factors such as wind speed [16], temperature [17], and sampler configurations [3,18], which makes it challenging to accurately convert time-integrated concentrations (mass/sampler) into airborne concentrations (mass/m^3^) [18,19]. The collection efficiencies of airborne particles also differ depending on the design of passive sampling chambers [9]. In addition, typical sampling rates could vary depending on sampling sites and seasons [17,20]. The calibration of the passive aerosol samplers for individual analytes is required using active samplers for long-term running to obtain the passive uptake rate of each target chemical (volume of air per day) [21,22]. Passive sampling of non-volatile species could be challenging depending on low particle diffusion [23]. 66.7% of PM_2.5_ collected by passive samplers could agree well with that collected by active samplers, while 93.3% of PM_10_ results were within the ±30% range determined with active samplers [24], indicating the particle size-dependent collection efficiency of passive sampling. In this regard, passive samplers provide the semi-quantitative analysis of atmospheric concentrations, which are inadequate for some applications such as occupational health requiring robust sampling protocols and insensitivity to environmental conditions [7]. Therefore, an alternative way similar to active sampling (known sampling rates and size-related collection efficiencies) without artificial power supply will further extend the acceptance and applications of passive samplers with quantitative sampling protocols.

A limited number of concepts of passive samplers, such as flow-through air samplers [25,26], have been developed to advance the quantitative sampling of airborne pollutants in a passive way. For example, a flow-through sampler has been developed to collect semivolatile organic compounds heavily relying on wind speeds, which works similarly to an active sampler with known sampled air volume measured by an anemometer [27]. Nonetheless, current flow-through passive samplers with large porosity are mainly suitable for sampling gaseous pollutants such as semivolatile organic compounds and nitrogen dioxide. The flow-through passive sampler works well for gases because of their diffusion rates, while the particle flux based on diffusion is weak depending on particle sizes [28]. Generally, the flow-through airflow is very sensitive to material porosity, which is associated with pressure drop. Any notable pressure drop will cause the air flow to bypass, and some feasible options for the flow-through passive samplers with large porosity could be single-layer wire mesh or some other materials with similar structures. The flow-through passive samplers with this large porosity will lead to a very low collection efficiency and collection amount of airborne particles. To the best of our knowledge, the flow-through sampler for airborne particles is still not available.

Light-driven aerogel passive pumps with a surface layer of the optical absorber can command a measurable passive gas flow induced by photothermal conversion. They can be tuned by the pore structure of aerogel materials, demonstrating their potential in air quality monitoring and control [29]. The thermal transpiration gas pump with a low thermal conductivity creates a thermal gradient field to enable an induced gas flow through porous materials, which have pore diameters approaching the mean free path length of the gas molecules (66 nm for air molecules) [30]. Polyimide (PI) aerogel has low thermal conductivity, high flexibility, and foldability [31], and air filtration is one of the promising applications due to the hierarchical porous structure and large specific surface area, which could achieve a filtration efficiency close to 100% [[32], [33], [34], [35]]. To achieve the thermal transpiration gas pump using PI, an optical adsorption layer could be created using a thermally decomposed PI layer, inducing the light-driven thermal gradient (Fig. 1a). Therefore, this PI aerogel could create a light-driven air flow and capture airborne particles carried by the flow, which could be an ideal dual-functional material working as the thermal transpiration gas pump for aerosol sampling.

**Fig. 1:**
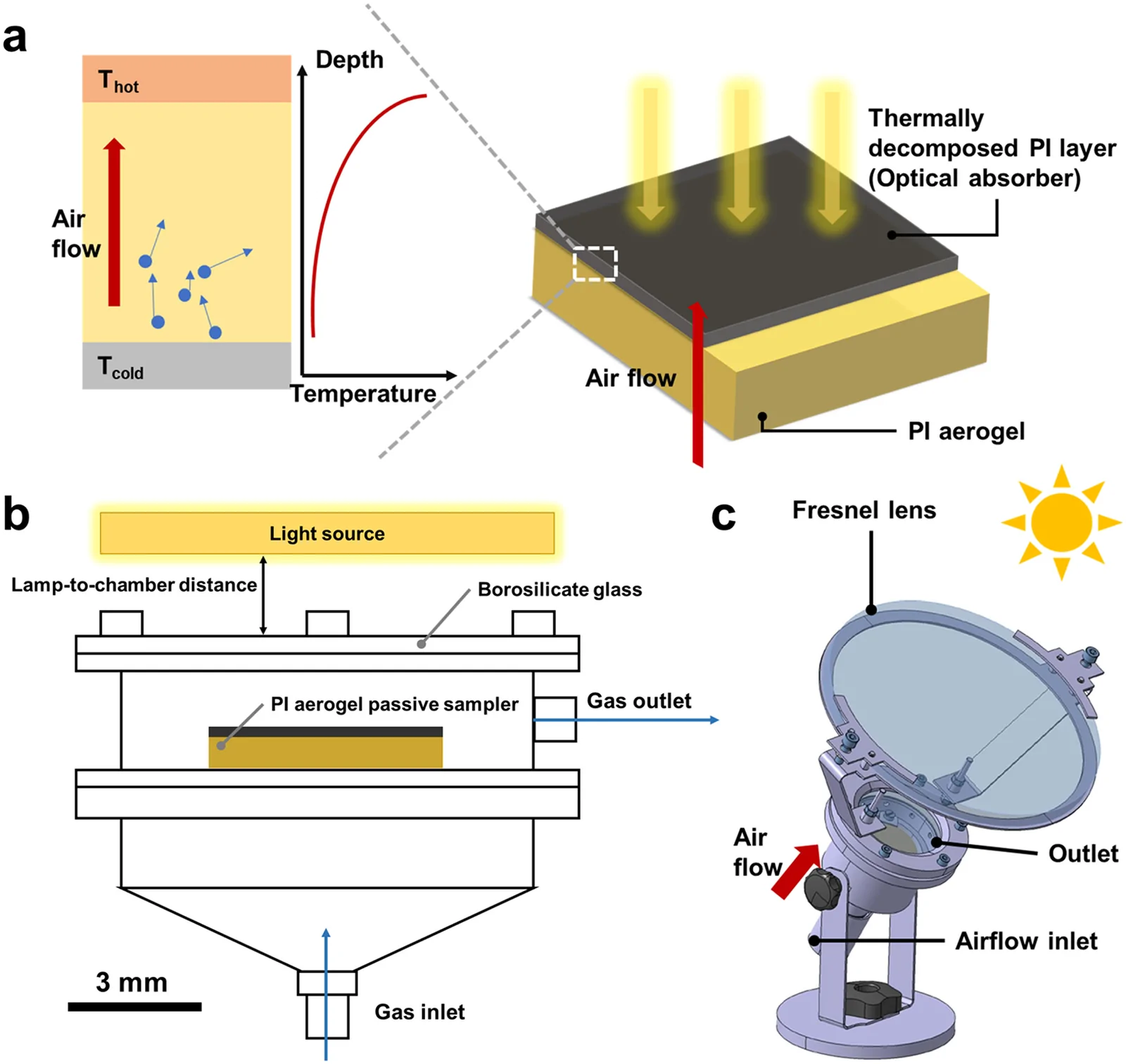
(a) Schematic illustration of the light-induced airflow using the PI aerogel passive sampler. (b) Schematic of the sampling chamber with glued PI aerogel passive sampler for particle sampling tests (with the gas flow going from the bottom to the top of the PI aerogel). (c) The prospective operation setup with the PI aerogel passive sampler employing exclusively the light-driven airflows for airborne particle collection in the real-world environment (The thermally decomposed PI layer is the optical absorber).

The conventional passive sampler is an aerosol sampler without an artificial power supply to use collection mechanisms such as gravitational settling and diffusion, while the active samplers require an additional power supply and collect airborne particles based on mechanisms such as filtration and impaction [28]. In this study, we develop a light-driven PI aerogel-based sampler in an active sampling-like way based on filtration. As this PI aerogel-based sampler does not need an artificial power supply, we would like to consider it a passive sampler. The polyimide aerogels with different pore sizes and structures were engineered and tuned to realize thermal transpiration due to temperature gradients, which were created by the thermally decomposed PI layer as the optical absorber (Fig. 1). The polyimide aerogel served as a light-driven gas pump and air filter to capture airborne particles. A sampling campaign in the ambient environment was conducted to evaluate the sampling performance of the PI aerogel, and particle morphology and water-soluble metals were analyzed. In future applications, a solar concentrator should be designed to efficiently concentrate sunlight for a relatively high light-driven sampling flow rate.

## 2 Materials and methods

### 2.1 Fabrication of the PI aerogel passive samplers

Poly (amic acid) precursor was made from the polycondensation of two diamines ODA (4, 4′-oxydianiline, Sigma-Aldrich) and DMBZ (m-tolidine, TCI chemicals) (molar ratio = 5:5), and dianhydride BPDA (biphenyl-3, 3′, 4, 4′-tetracarboxylic dianhydride, Sigma-Aldrich) (Fig. 2). Specifically, the order of the monomers to synthesize poly (amic acid) is ODA, BPDA, and DMBZ to get alternate copolymerized oligomers as shown in previous work [36]. In this work, the repeating unit of poly (amic acid) oligomer is 30, realized by controlling the molar ratio of diamine (ODA and DMBZ in total) and dianhydride at 31:30. The diamine is excess to the dianhydride, resulting in amino group terminated poly (amic acid) oligomers, considering the crosslinker of BTC (1, 3, 5-benzene tricarboxylic acid, Sigma-Aldrich). The homogeneous mixture was transferred into silicon molds and the gelation occured within 5 to 15 min, depending on the detailed recipes. Obtained wet gels were aged for 24 h at room temperature, and the solvent was exchanged with 75/25, 25/75, and 0/100 NMP (N-methyl-2-pyrrolidone, Chemie Brunschwig AG) (or DMAc (dimethylacetamide, Chemie Brunschwig AG)) /ethanol (5% isopropanol, Alcosuisse) in 12 h intervals. The alcohol gels were dried under supercritical CO_2_ (SCF extractor, Separex) (120 bar, 50 °C). Additionally, the above-mentioned poly(amic acid) was fabricated in different solvents, including 100 vol% NMP, 50/50 vol% NMP/DMAc, and 100 vol% DMAc.

**Fig. 2:**
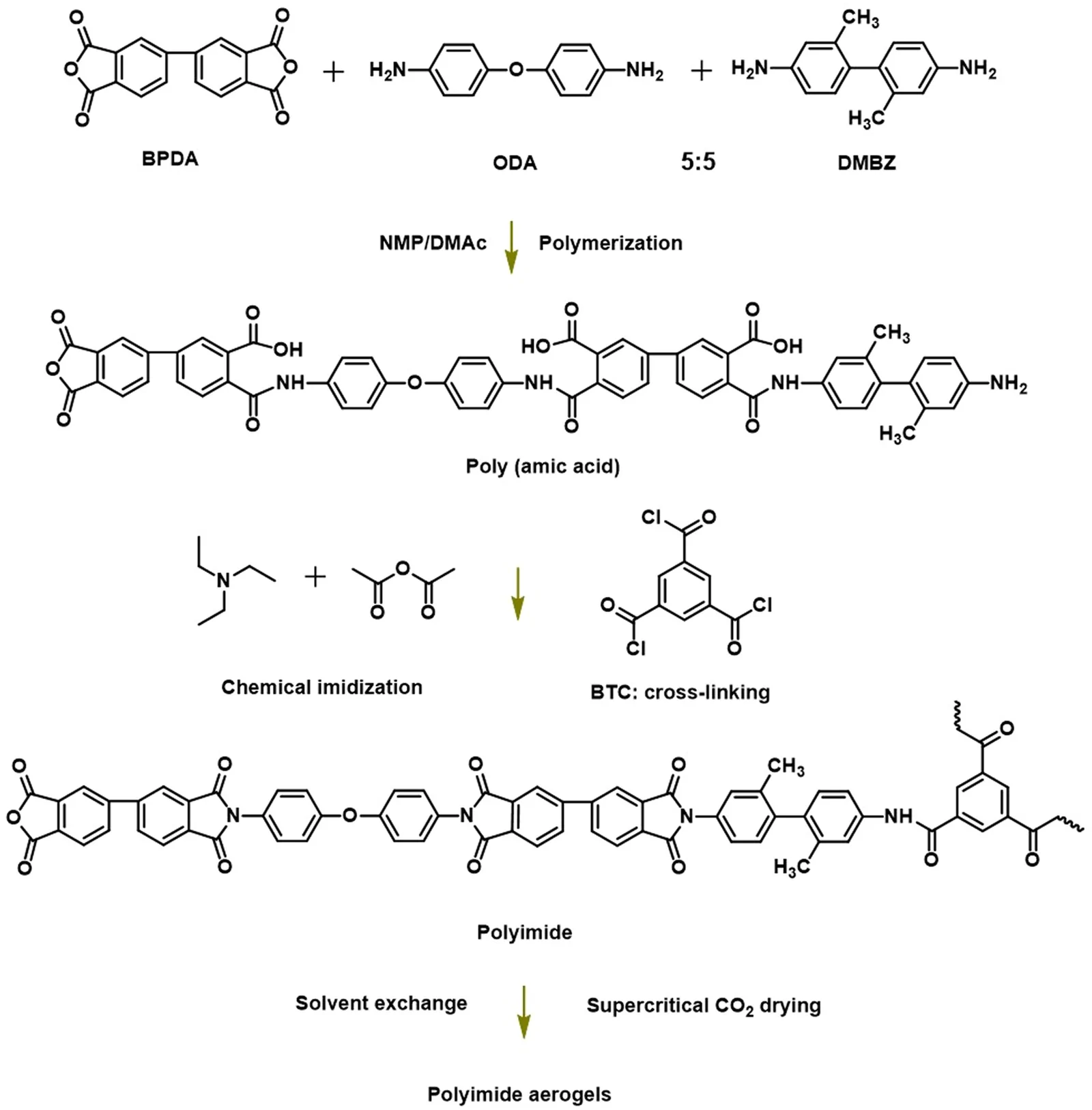
**Synthesis of the PI aerogel**.

A detailed preparation process for the 50/50 vol% NMP/DMAc system is presented as follows. 25 mL NMP and 25 mL DMAc were premixed before the polymerization. 0.7453 *g* ODA was firstly dissolved in 10 mL 50/50 vol% NMP/DMAc for 5 min, and then 2.1196 *g* BPDA was added for stirring 5 min with a mechanical overhead stirrer [37,38]. Afterward, 0.7902 *g* DMBZ was added to the systems. The whole mixture was stirred for 30 min to get a poly(amic acid) oligomers solution. Afterward, 5.448 mL acetic anhydride (Lonza) and 8.033 mL TEA (trimethylamine, Sigma-Aldrich) were premixed and then added into obtained poly (amic acid) solution for 1–3 min. Lastly, 0.0425 *g* BTC was first dissolved in 10 mL 50/50 vol% NMP/DMAc and then added into poly (amic acid) solution for 1–3 min. The total polymer concentration of the system was kept at 7 wt%.

The polyimide (PI) aerogels were named mesoporous, hierarchical, and spherical (based on their microscope structures, as shown in Fig. 3a, b, and c) with a thickness of 5 mm, respectively. The thickness of the carbonized PI layer was kept as thin as possible (around 0.5 mm) due to space constraints, to ensure the maximum thickness and temperature difference between the top carbonized PI layer and the bottom layer, resulting in the maximum photothermal effect and light-driven flow rates. A combination of spherical (thermally decomposed PI layer side) and hierarchical (particle sampling side) was prepared to have the best performance of light absorption (from spherical) and porous structure (from hierarchical), which was named spherical/hierarchical. The effects of pore size and structure were investigated by comparing the temperature differences and flow rates among hierarchical, spherical, and spherical/hierarchical.

**Fig. 3:**
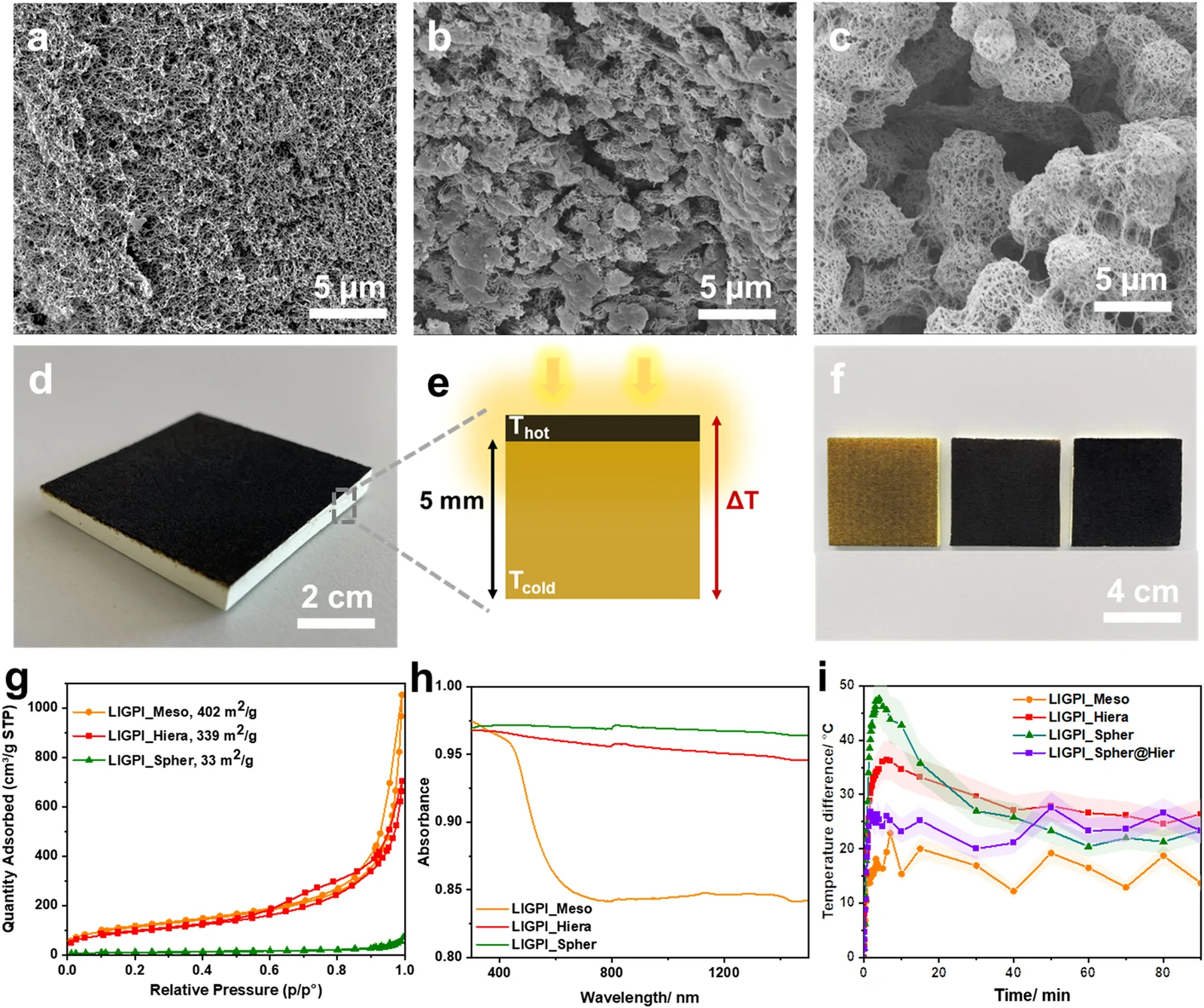
**Morphology and light-to-heat conversion of the PI aerogel passive sampler.** Cross-section SEM images of the sample (a) mesoporous, (b) hierarchical, and (c) spherical. (d) Side view of the hierarchical aerogel sample and (e) schematic illustration of the temperature difference induced by light across the PI aerogel. (f) Photos of the three types of PI aerogel passive pumps (from left to right: mesoporous, hierarchical, and spherical). (g) BET specific surface area testing of three aerogel passive pumps. (h) UV–vis adsorption of different light wavelengths of three types of aerogel pumps. (i) Temperature difference between the hot and cold sides of different PI aerogels with a lamp-to-chamber distance of 20 cm.

### 2.2 Performance evaluation of the PI aerosol passive samplers

The PI aerogel passive sampler with an effective diameter of 3 cm for air sampling was glued covering a hole (*Ø* = 3 cm) of a metal plate using a polysiloxane-based adhesive (Elastosil RT 601 A and B, Wacker AG), which was integrated into a sampling chamber with a borosilicate glass window on the top (Fig. 1b). An artificial light source (halogen lamp, 300 W) was used to obtain a consistent light intensity, which was tuned by adjusting the lamp-to-chamber distance, and distances of 20 cm and 5 cm away from the light source were equivalent to the light intensity of one sun and two suns, respectively (Fig. 1b). The absorbance of the optical absorption layer was measured using UV–Vis spectroscopy (UV-3600, Shimadzu) in the wavelength range of 300–1500 nm. The collection efficiency, flow rate, and temperature difference between the top and bottom layers of mesoporous, hierarchical, spherical, and spherical/hierarchical were measured (Fig. S1). The collection efficiency of particles was determined by using a scanning mobility particle sizer (SMPS, consisting of a Differential Mobility Analyzer (DMA, model 3080, TSI) and Condensation Particle Counters (CPC, model 3775, TSI)) and an aerodynamic particle sizer (APS, model 3321, TSI) (Fig. S1a). 1% NaCl solution was used in a homemade atomizer to generate airborne NaCl particles with a flow rate of 2.8 L/min, and a 60 L plastic container was employed as a mixing chamber to maintain a quasi-equilibrium of aerosol NaCl as the aerosol source for evaluating collection efficiency. The typical NaCl particle number ($C_{up}$) of smaller than 650 nm and larger than 550 nm was about 2.7 × 10^6^ and 3200 particle/cm^3^ in the plastic mixing chamber, obtained by SMPS and APS, respectively (Fig. S2), which could be 1000 times higher than the ambient environment to ensure a sufficient particle number concentration for the particle test. A filtered flow was introduced into the downstream as the makeup flow for downstream particle counting, as the working flow of SMPS (0.3 L/min) and APS (1 L/min) was much higher than that of the PI aerogel passive sampler (< 10 mL/min). The downstream particle number concentration was measured with the artificial light on and off, and the difference between particle numbers with the light on and off was the actual downstream particle number concentration ($C_{down}$) caused by the PI aerogel serving as the light-driven gas pump, which was diluted by the makeup flow. Note that the downstream particle number with the light off should be close to zero, indicating no air leakage during collection efficiency measurements. The dilution factor (D) was defined as the ratio of the working flow of SMPS or APS to the gas flow of the PI aerogel passive sampler. Therefore, the downstream particle number concentration passing through the PI aerogel passive sampler should be the measured downstream particle number multiplied by the dilution factor. The collection efficiency (η) was calculated based on the following equation:

$$\eta =\;\frac{C_{up}-C_{down}\cdot D}{C_{up}}$$

For the flow rate measurements, a closed loop with the sampling chamber and flowmeter (MFC, AALBORG TIOTotalizer) was used with the artificial light on (Fig. S1b). The temperature kinetic experiment was conducted using two temperature sensor tips (Type T, Cu-CuNi or RS PRO RS-172TK) contacting the surface of the top and bottom layer (Fig. S1c). The cross-section morphology of the PI aerogel passive sampler and the particle penetration were investigated using scanning electron microscopy (SEM), and the composition of collected particles was analyzed by energy dispersive X-ray spectroscopy (EDS) [39]. The samples were coated with a layer of 15 nm Pt (measured with a flat piezo detector) to avoid charging before the measurement.

The hierarchical PI aerogel (4 cm × 4 cm × 0.5 cm) with a weight of 740 mg was used in the real-world application (i.e. it was exposed to ambient aerosol), which exhibited the highest flow rate among PI aerogels with different pore sizes (mesoporous, hierarchical, spherical, and spherical/hierarchical). For the composition analysis of sampled particles, three aerogels served as the light-driven aerogel (aerosol sampling with light onto it), control aerogel (aerosol sampling without light onto it), and blank aerogel (without collected airborne particles for analytical measurements), respectively. For the light-driven aerogel, the top side and bottom side worked as the surface for conventional passive sampling (including gravity and diffusion-driven sampling) and light-driven sampling, respectively. The sampling campaign was conducted with the artificial lamp (lamp-to-chamber distance: 20 cm) on the balcony of the Empa laboratory building in Dubendorf, Switzerland for 24 days (May 20 – June 13, 2023). An active sampling filter (0.45 µm cellulose nitrate filter, Sartorius Biotech) with a flow rate of 5.2 L/min was used to estimate the average airborne metal concentration. Prior to the determination of water-soluble fraction, samples were ultrasonically extracted with Milli-Q water (18 MΩ) for 30 min, and subsequently filtered using 0.2 µm syringe filters [40]. The extractants were acidified with 1% (v/v) HNO_3_ before ICP-MS analysis. The size-resolved information of airborne particles in the ambient environment was obtained by performing a sampling campaign using Nano MOUDI 122NR impactor (with the cut-sizes of 10,000, 5600, 3200, 1800, 1000, 560, 320, 180, 100, 56, 32, 18, and 10 nm at a sampling flow rate of 30 L/min [41]) for 5 days and 10 h (during July 20–July 30, 2021) and subsequent ICP-MS analysis. The morphology of the aerogel samples and elemental distribution were investigated using SEM/EDS.

## 3 Results and discussion

### 3.1 Sampling performance of the PI aerogel passive sampler

The color of the optical absorption layer was dependent on pore size and structure, and the PI aerogel with larger pore sizes tended to maintain its original structures after being thermally decomposed by laser, resulting in a dark black color (Fig. 3d, 3e and 3f). A thickness of 5 mm enabled the distinct temperature difference between the top and bottom layers (Fig. 3e). The specific surface area of the mesoporous, hierarchical, and spherical aerogel was 402, 339, and 33 m^2^/g, respectively (Fig. 3g). The spherical/hierarchical sample was a physically combination of a half spherical and half hierarchical layer. Therefore, the surface area of the spherical/hierarchical sample was the average of the specific surface areas of the spherical and hierarchical samples. In this case, the sample-specific surface area decreased in the order of mesoporous > hierarchical > spherical/hierarchical > spherical. As shown in Fig. 3h, the dark black hierarchical and spherical aerogel exhibited a stronger light absorption (> 0.95) in the wavelength of 300–1500 nm, whereas the aerogel with the mesoporous structure showed a lower absorbance (< 0.85) in the range of 600–1500 nm. The temperature difference between the optical absorption layer and PI aerogel layer suggested that the mesoporous sample with a weaker light absorption resulted in a lower temperature gradient, compared to the hierarchical and spherical aerogel (Fig. 3i). In particular, the temperature difference of the mesoporous, hierarchical, spherical, and spherical/hierarchical aerogel after the light exposure for 30 min was 15.73 ± 2.81, 26.91 ± 1.59, 23.31 ± 2.38, and 23.64 ± 2.72 °C, respectively.

Airborne particles carried by light-driven airflow were mainly captured on the low-temperature side (Fig. 4a). The initial flow rate was increased rapidly as a result of the thermal expansion of the gas in the sampling chamber, and the stabilized flow rate was induced by thermal transpiration [30]. The steady-state flow rate after 30 min for mesoporous, hierarchical, spherical, and spherical/hierarchical aerogel was 2.73 ± 0.05, 4.92 ± 0.05, 1.70 ± 0.04, and 3.02 ± 0.10 mL/min, respectively (Fig. 4b). According to SEM images (Fig. 3a, 3b, and 3c), the fraction of macroporous pores for different PI aerogels could be spherical > hierarchical > mesoporous. Therefore, the hierarchical sample with a mixture of mesoporous and macroporous pores allowed the highest flow rate, suggesting that a moderate fraction of macroporous pores (lower than that in spherical aerogels) favored higher air flow by providing a larger porosity. The spherical/hierarchical aerogel consisted of spherical particles for optical absorption and the hierarchical structure for smaller pore sizes and favorable gas transport, and the thermal-induced flow rate for the hierarchical sample was higher than that for the spherical/hierarchical aerogel (Fig. 4b). That is, the replacement of half hierarchical structure with a spherical structure (larger pore size) caused a lower flow rate, indicating that the pore size and structure had significant effects on the flow rate. Pore size and temperature differences had affected the flow rate collectively, and pore size might play a dominant role as hierarchical and spherical samples with similar light absorption exhibited the highest and lowest flow rates, given the same light intensity.

**Fig. 4:**
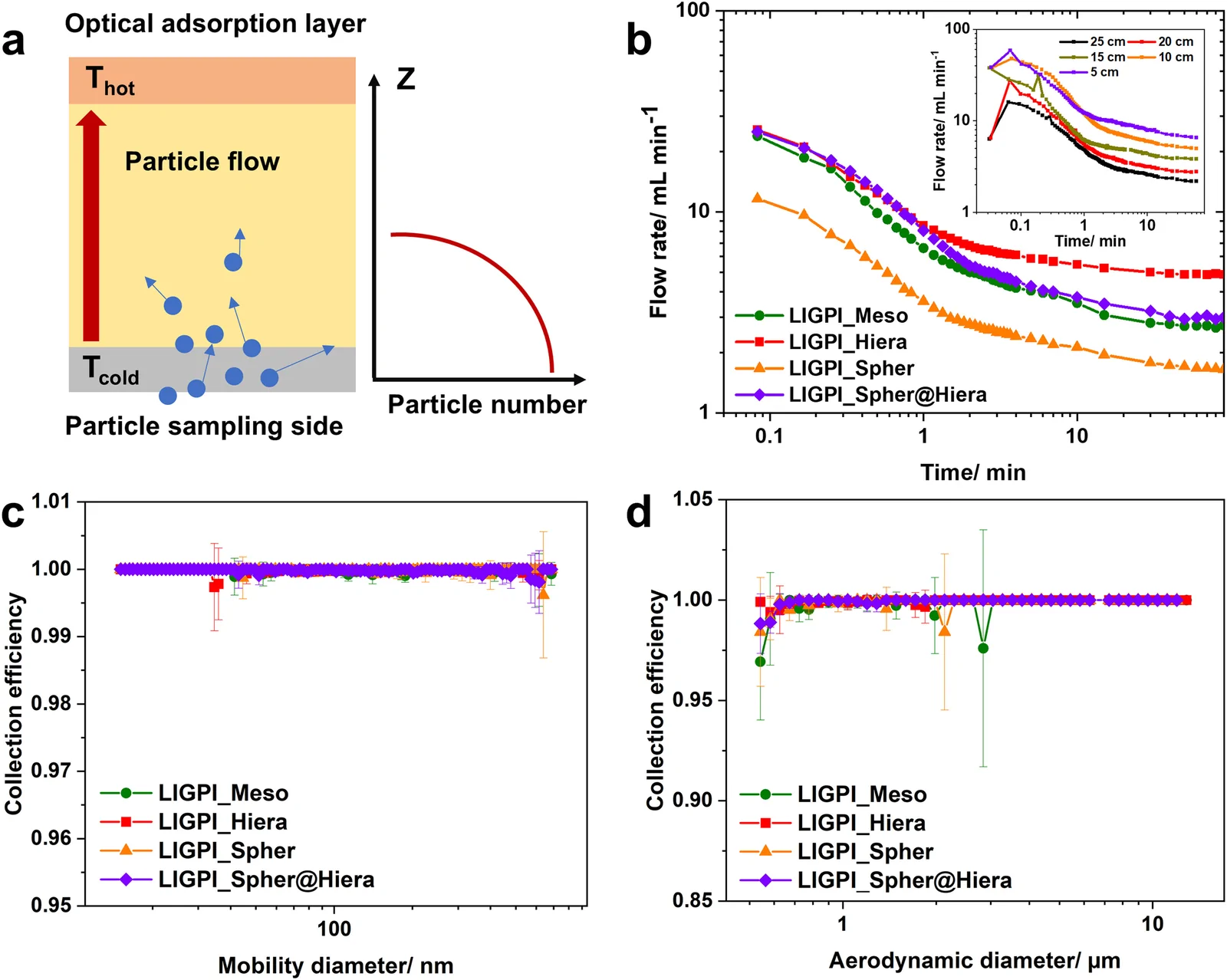
**Airborne particle sampling using the PI aerogel pumps.** (a) Schematic of the light-driven airborne particle capture. (b) Sampling flow rates (effective area: 7.07 cm^2^) for different porous structures of aerogel, and the inset plot is the light-driven flow rates of the spherical/hierarchical PI aerogel as a function of the lamp-to-chamber distance. Collection efficiency was measured by (c) SMPS and (d) APS of the PI aerogel passive pump with a lamp-to-chamber distance of 20 cm.

There was a clear trend that the flow rate increased and then decreased with increasing pore sizes, which suggests that the optimal pore size and structure is close to that of the hierarchical sample showing the highest flow rate. For the spherical/hierarchical aerogel, it exhibited an increasing airflow rate with short lamp-to-chamber distances, which provided greater temperature gradients (Fig. 4b). Mesoporous, hierarchical, spherical, and spherical/hierarchical aerogel exhibited a sampling efficiency close to 100% in a broad particle size range of 20 nm–10 µm (Fig. 4c and d). The sampling rates in a flow-through mode and collection efficiencies (∼100%) demonstrated that the PI aerogel passive sampler enabled the sampling in an equivalent way of active sampling (with sample flow rates measured, e.g. by an anemometer after scaling up the flow rate by increasing aerogel face areas) and quantitative analysis of airborne particles, unlike the conventional passive samplers in need of sample rate calibration assisted by active samplers during sampling periods for semi-quantitative analysis.

### 3.2 Distribution of sampled NaCl Particles

The penetration and distribution of NaCl particles across the PI aerogel passive samplers were investigated (Fig. 5). It was found that the penetration of NaCl particles depended on the pore size and structure of the PI aerogel. No NaCl particle was observed in the cross-section of the mesoporous aerogel (in the direction of the aerogel thickness), and the sampled NaCl particles including submicron particles were homogenously distributed on the sampling surface (Fig. 5a). In contrast, more NaCl particles penetrated the PI aerogel with larger pore sizes (hierarchical and spherical aerogel) (Fig. 5b and c). For the cross-section view at a larger scale, spherical aerogels showed a more porous structure than mesoporous and hierarchical samples (Fig. 5d, 5e, and 5f). EDS elemental mapping also confirmed no Na penetration through mesoporous and hierarchical samples (Fig. 5, Fig. 5), while Na penetrated the spherical aerogel within 100 µm (Fig. 5i). Interestingly, the uneven distribution of NaCl particles was observed on the surface of the hierarchical aerogel (Fig. 5c), which suggested that the gas flow paths through the hierarchical aerogel were distributed heterogeneously compared to mesoporous PI aerogels. The agglomeration of NaCl particles collected on the aerogel surface shown in Fig. 5c was likely due to the long residence time of NaCl particles around the inlet of the sampling chamber, which should be optimized in real-world applications. In the current sampling chamber, the inlet volume could be around 1 L, and the residence time could be more than one hour, given a light-driven flow rate of 1–10 mL/min. In addition, humidity could affect the shape and size of the NaCl particles during their suspension in the air.

**Fig. 5:**
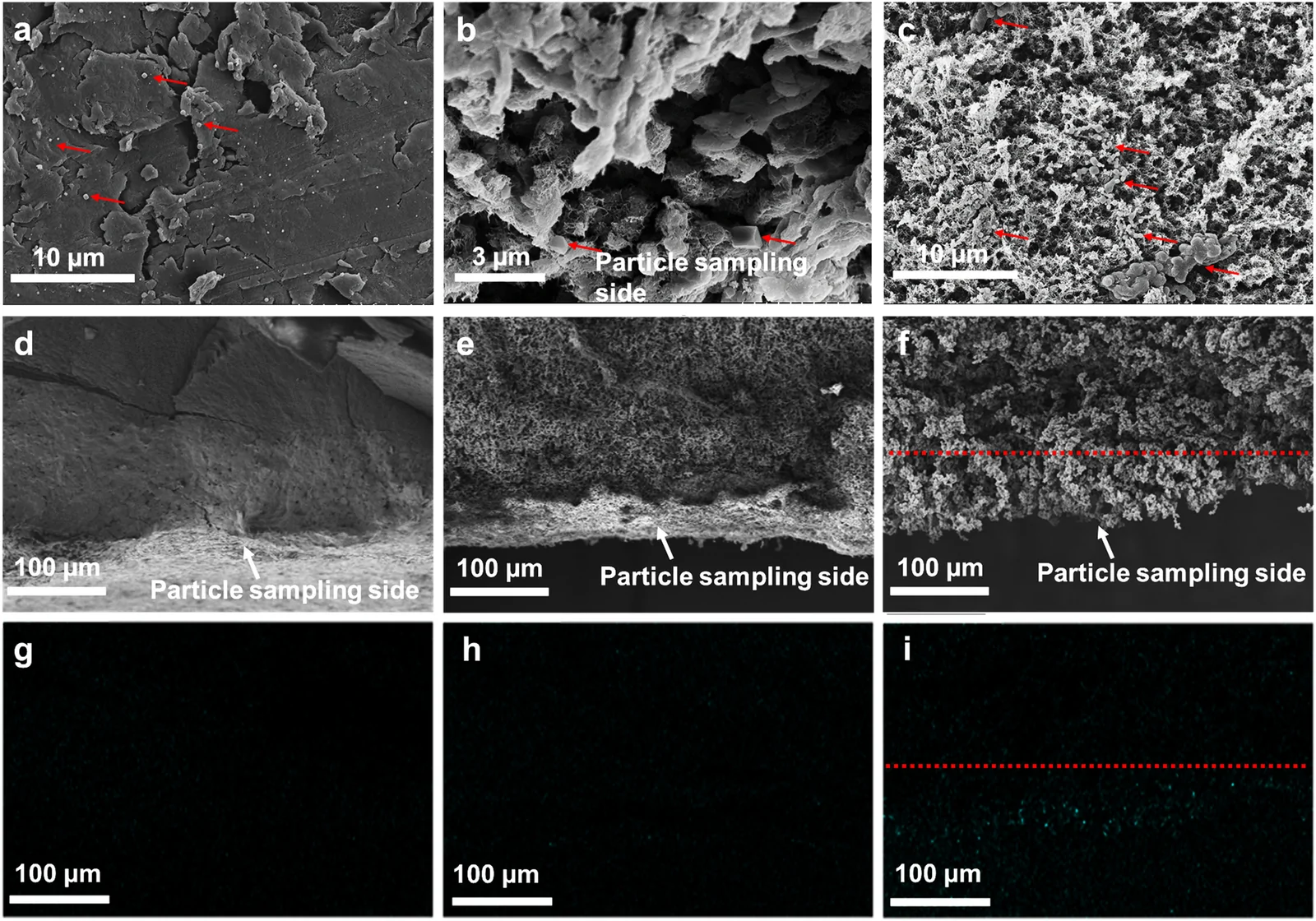
**SEM images of PI aerogel passive pumps after airborne NaCl particle collection for 6 h.** Mesoporous (a) surface (red arrows indicated the examples of NaCl particles). Hierarchical (b) cross-section and (c) surface. Cross-sectional SEM images of the (d) mesoporous, (e) hierarchical, and (f) spherical sample after NaCl particle collection for 6 h with the light on (lamp-to-chamber distance: 20 cm), and the corresponding EDS mapping of Na (g, h, and i). Na mapping was not visible in Fig. 5g and 5h, indicating few NaCl particles penetrating the PI aerogel vertically. The red dashed line in Fig. 5f and 5i indicated the upper limit of Na distribution (blue points) in the sample cross-section.

### 3.3 Effects of light intensity and pore size on sampling performance

The light intensity significantly affected the temperature gradient between the top (the optical absorption layer) and bottom layer, which directly impacted the thermal-induced flow rate. In general, a stronger light intensity led to a higher temperature difference (Fig. S3), and subsequently caused a higher flow rate (Fig. S4). The relationship between the temperature difference and flow rate suggested that pore size and structure played a dominant role in how the flow rate responded to temperature differences (Fig. 6). Given the same temperature difference (e.g. 25 °C), the hierarchical aerogel induced the highest flow rate, which features mesoporous and microporous gas paths. The combination of mesoporous and microporous pores also favors a more sensitive response of airflow rate to the temperature difference (Fig. 6). With different light intensities, mesoporous, hierarchical, spherical, and spherical/hierarchical samples maintained a collection efficiency higher than 80% in the particle size range of 100–650 nm and 800 nm–10 µm according to the SMPS and APS results (Figs. S5 and S6). The spherical aerogel sample with the largest pore sizes exhibited a slightly lower collection efficiency of small particles (e.g. 100 nm) (Fig. S5). Other factors affect collection efficiencies, such as thickness [42]. The thickness of the PI aerogels was around 5 mm, which might play a major role in the collection efficiency over porous structures. Note that the lower collection efficiency of particles smaller than 1 µm (mainly 70–200 nm) was partially due to the released particles from the optical absorption layer caused by high temperatures up to 150 °C (with a lamp-to-sampler distance of 5 cm) (Figs. S6 and S7), which agrees with the previous study showing a weight loss of 3 wt% below 100 °C [43]. The particles released from the carbonized PI layer (filter downstream) increased the downstream particle number concentration and could underestimate the collection efficiency. A lamp-to-sampler distance of 20 cm could provide a light intensity equivalent to that of one sun, which could be more suitable for simulating normal day conditions. In addition, a smaller lamp-to-sampler distance could cause the release of airborne particles from the carbonized aerogel surface (Fig. S7), which might be caused by slight decomposition of carbonized aerogel surface at a high temperature. Therefore, a lamp-to-sampler distance of 20 cm was chosen in the study.

**Fig. 6:**
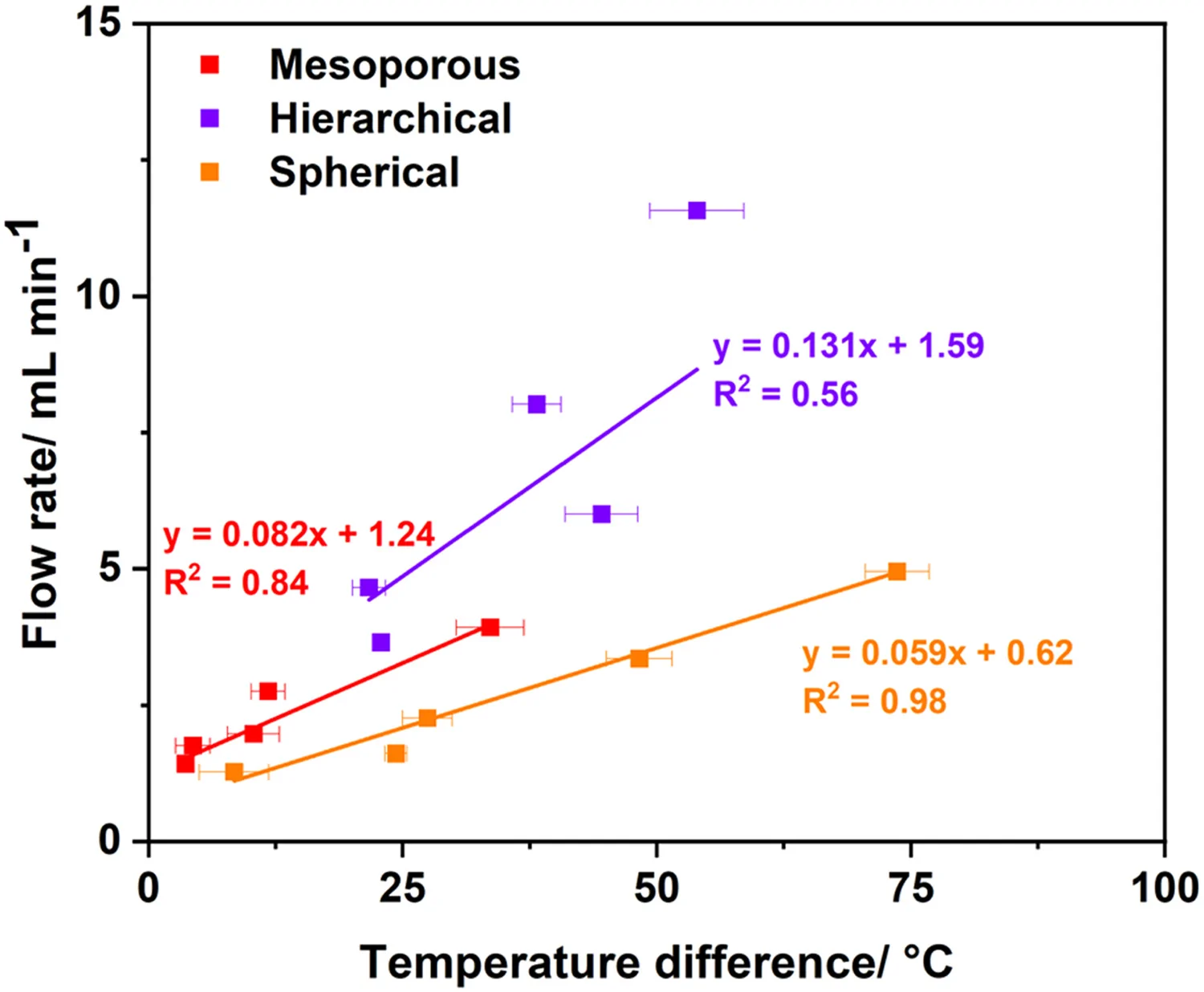
Relationship between the average temperature difference (light on for 30 min) and sampling flow rates (light on for 30 min) using the mesoporous, hierarchical, and spherical aerogel passive samplers.

### 3.4 Sampling of airborne particles in the real-world environment

The light-driven hierarchical aerogel was employed in the real-world environment to collect ambient samples (Fig. 7a). SEM images showed that submicron airborne particles were collected on the porous structures of the PI aerogel passive sampler, solid and sphere-like, as indicated by the red arrows (Fig. 7b). The penetration of ambient airborne particles could be effective within 15 µm in depth (Fig. 7c). EDS elemental mapping exhibited relatively uniform distribution of carbon and oxygen in the cross-section of the original hierarchical aerogel (Fig. S8b and S8c), while local enhanced signals of carbon and oxygen were shown after collecting ambient particles (Fig. S8e and S8f), indicating the sampling of carbon-rich and oxygen-rich ambient particles. Many of the airborne particles smaller than 1 µm were collected on the surface of the PI aerogel passive pump (Fig. 7d and e). Due to the relatively low ambient particle concentrations (around 3000 particle/cm^3^) and sampling flow rates (around 5 mL/min), the number of collected particles was limited to around 212 particle/min cm^-2^, which explains the limited number of observed particles captured on the PI aerogels. Given the sampling flow rates, the residence time of airborne particles could be significant for particle agglomeration inside the inlet of the sampling chamber used for the PI aerogel passive samplers, which accelerated the particle deposition downwards at a low flow rate due to their gravity effect, and subsequently reduced the airborne particle number for sampling.

**Fig. 7:**
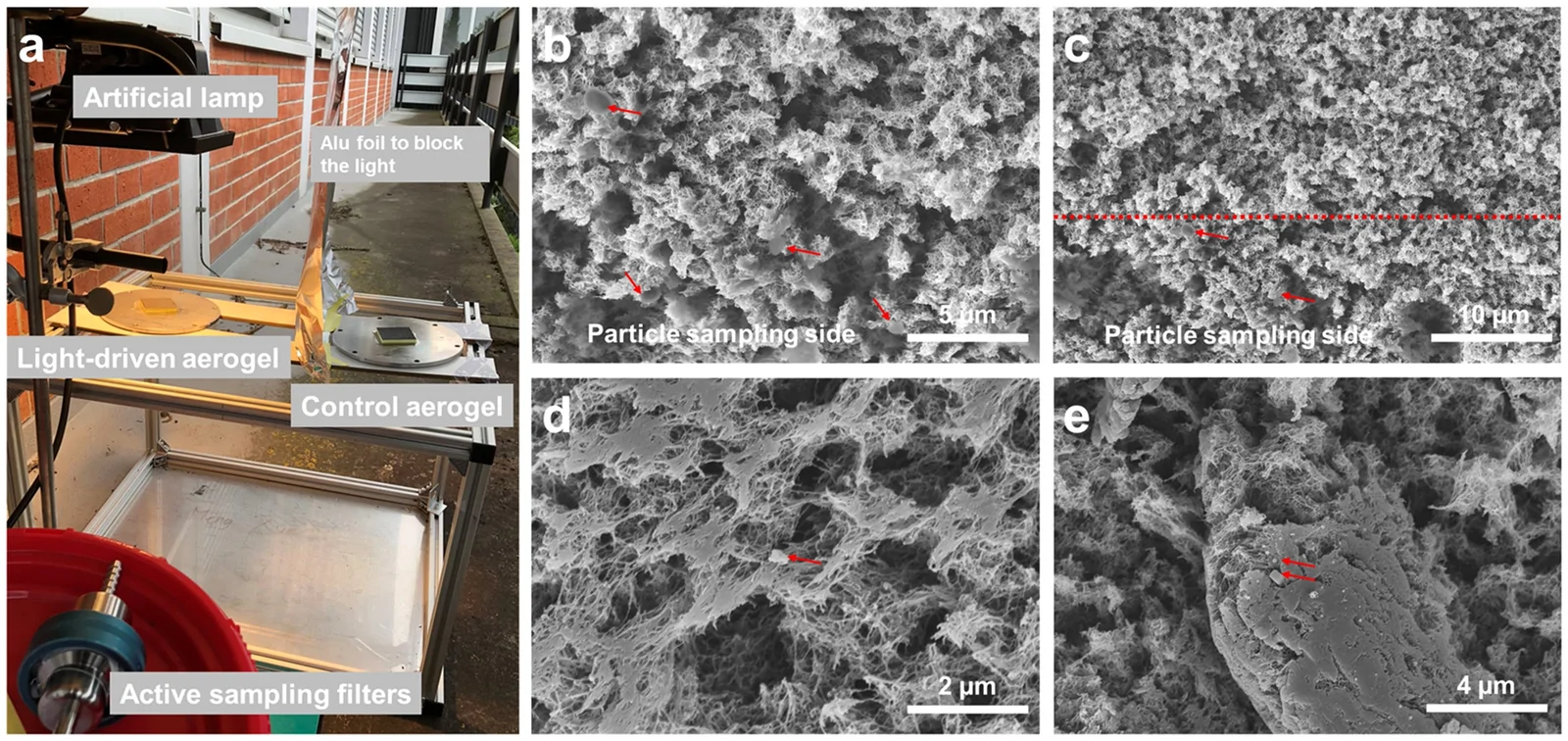
**Application of the light-driven hierarchical aerogels in the ambient environment for water-soluble metal analysis (a).** SEM images of the hierarchical PI aerogel passive sampler after ambient particle collection over one week with artificial light on (lamp-to-sampler distance: 20 cm). SEM images of the PI aerogel passive sampler cross-section (b) and (c), and surface (d) and (e). The red arrows indicated the presence of ambient particles, and the red dashed line indicated the upper limit of particle penetration through the PI aerogel passive sampler.

Analysis of water-soluble metals showed that the sampling flow rates of the light-driven and control PI aerogel were similar in terms of water-soluble metals such as Mg and Fe (Table 1). The concentration difference of Mg in the light-driven and control samples might be caused by the size-dependent Mg concentration, and water-soluble Mg was more concentrated on the airborne particles larger than 1 µm (Fig. S10b), which could be subject to many environmental factors. Other metals, such as Fe, Cu, Zn, and Pb, were more concentrated in particles smaller than 1 µm (Fig. S10). In comparison, the concentration of heavy metals including Cu and Zn sampled by the light-driven PI aerogel, was much lower than that collected by the PI aerogel without light on (Table 1). It is likely due to the increasing adsorption of heavy metal ions at high temperatures on the PI aerogel or airborne particles after long-term light and thermal exposure. It is noted that the light-driven flow rate (assuming 5 mL/min) could be 32–128 times lower than the combination of passive sampling rate and light-driven flow rate (160–640 mL/min) (Table 1). The lower metal concentration collected on the light-driven sample compared to the control passive sample as shown in Table 1, indicated that thermophoresis might play an important role in collecting airborne particles, which push particles towards cold sources in a temperature gradient [44]. More specifically, fewer particles were passively collected on the hot aerogel surface compared to conventional passive sampling. Conventional passive sampling relies on size-sensitive gravitational settling and diffusion, while the proposed light-driven Knudsen pump introduces additional thermophoresis and light-driven airflow for collecting particles. Due to the low light-driven flow rate, further scale-up experiment is needed to investigate the interaction between the conventional passive sampling and light-driven sampling. Conventional passive sampling based on particle deposition and diffusion could be excluded from aerosol sampling by covering the PI aerogel with a glass on the top of the sampling chamber (Fig. 1c), so that light-driven sampling dominates in sampling campaigns.

**Table 1:** **Analysis of water-soluble metals in ambient particles collected by the hierarchical PI aerogels**.

	Mg	Fe	Cu	Zn	Pb
Airborne concentration (ng/m^3^)^1^	56.69 ± 0.22	17.13 ± 0.19	5.62 ± 0.03	21.19 ± 0.25	0.17 ± 0.002
Equivalent sample flow rate for the light-driven sample (L/min)^2^	0.64 ± 0.01	0.16 ± 0.01	0	0.09 ± 0.002	0
Equivalent sample flowrate for the control sample (without lamp on) (L/min)^2^	0.85 ± 0.003	0.17 ± 0.01	0.13 ± 0.004	0.26 ± 0.004	0

1 The airborne concentration was calculated based on the concentration of collected water-soluble metals using active sampling.

2 The equivalent sample flowrate = total mass of water-soluble metals collected by the light-driven or control sample (ng)/that sampled by the active sampling next to the light-driven and control sample (ng)*active sampling flowrate (5.2 L/min).

### 3.5 Limitations and future outlooks

To measure the collection efficiency, the low light-driven flow rate will need filtered air to make up the high flow rates of the aerosol instruments such as Scanning Mobility Particle Sizer (SMPS) and Aerodynamic Particle Sizer (APS), which are 0.3 and 1 L/min, respectively. It suggested that we had to dilute the aerosol particle concentration by > 60 and 200 times, and the ambient particle concentration would be diluted (assuming 3000 particle/cm^3^) and result in a concentration of lower than 50 and 15 particle/cm^3^ respectively. The downstream particle number concentration would be even lower due to aerogel filtration, which could fall into the noise background of the aerosol instruments, and amplify the measurement deviation. The PI aerogels were tested using artificially generated particles similar to ISO 21,083–1 and ISO 16,890–1, which are widely used to characterize the filter performances. In our study, we measured the collection efficiency of the PI aerogels for > 10 min, and the collection efficiency remained unchanged (Fig. S11). It could be reasonable to conclude that the collection efficiency of the PI aerogel is approaching 100%. The measurement of collection efficiencies in real atmospheric conditions could be performed if the surface area of the PI aerogel is large enough to produce a high light-driven airflow rate at a level of L/min, which is also one of the key steps toward future field deployment.

The sampling air volume using the PI aerogel passive sampler can be measured by an anemometer [27], after scaling up the flow rate by increasing aerogel face areas, which allows quantification equivalent to active air sampling. However, the flowrate measurement suggested that the solar light-driven flow rate was at the mL/min level, which will result in about 10 – 100 folds lower sampling air volume compared to other passive aerosol samplers (e.g. with a sampling rate of 4 – 9 m^3^ per day (2.8 – 6.3 L/min) for trace metals [40]). This might hinder the sample analytical analysis without adequate particle mass based on PI-based aerogel passive samplers. A surface filtration type of filter as the sampling layer could be integrated into the PI aerogel passive sampler to limit the particle penetration, which will give rise to a sandwich-like structure consisting of an optical absorption layer (thermally decomposed PI aerogel layer), middle layer (PI aerogel), and sampling layer (thin and porous filter). Given the limited depth of particle penetration through the PI aerogel, the sampling layer could be collected for aqueous extraction and chemical analysis, while the other two layers could be reused with a new sampling layer. In addition, the thin and porous filter layer allows minimally treated samples for analytical analysis (e.g. Inductively Coupled Plasma Mass Spectrometry (ICP-MS) and Gas Chromatography Mass Spectrometry (GC–MS)) in the scale-up of one or multiple PI aerogel passive sampler arrays for maximal sampling air volume and particle mass.

Changing the solar incidence angle and sunlight intensity during a day will greatly affect the thermal-induced temperature and light-capture efficiency of the optical absorption layer, and light-induced sampling rate. A non-tracking solar concentrator with a trough shape [45] or a combination of large Fresnel lens and segmented mirrors [46] could be engineered to efficiently concentrate irradiation from all angles, which will be combined with the PI-based aerogel passive sampler to ensure the light-induced sampling rate measured by an anemometer during the day (Fig. 1c). The inlet of the passive sampler should be carefully designed (e.g. for multiple inlet openings and minimized inlet volume) to ensure the fresh and free-flow air quickly reaches the surface of the PI aerogel, which could eliminate the particle agglomeration and loss with a long residence time.

## 4 Conclusion

In this work, a light-driven PI aerogel passive sampler was proposed and tested, which was shown to have a high collection efficiency of airborne particles close to 100% and could operate at measurable flow rates, enabling quantitative analysis of airborne pollutants after passive sampling. The collection efficiencies using conventional passive sampling remain uncertain and are affected by various sampling and environment conditions, whereas the proposed light-driven PI aerogel passive sampler achieves nearly a 100% collection efficiency of airborne particles in nanometer to micrometer ranges with an active airflow equivalent to that typically created by active sampling, but without additional power supply during sampling campaigns. The pore size and structure could be further engineered to tune temperature gradients and flow rates of the passive pump, induced by sunlight based on thermal transpiration. The careful integration of the PI aerogel passive sampler into passive sampling devices should address the low sampling rate and changing light absorption in field studies by introducing a thin layer of particle sampling (to increase the sampling surface for a high sample air volume, and decrease the aerogel volume for sample preparation in a low extraction water volume and further analysis) and a non-tracking solar concentrator.

## Declaration of competing interest

The authors declare that they have no conflicts of interest in this work.

## Appendix Supplementary materials

Image, application 1

## References

[1] Daellenbach K.R., Uzu G., Jiang J. (2020). Sources of particulate-matter air pollution and its oxidative potential in Europe. Nature.

[2] Gu B., Zhang L., Van Dingenen R. (2021). Abating ammonia is more cost-effective than nitrogen oxides for mitigating PM2.5 air pollution. Science.

[3] Zhang X., Wong C., Lei Y.D. (2012). Influence of sampler configuration on the uptake kinetics of a passive air sampler. Environ. Sci. Technol..

[4] Marć M., Tobiszewski M., Zabiegała B. (2015). Current air quality analytics and monitoring: A review. Anal. Chim. Acta.

[5] McLagan D.S., Mitchell C.P.J., Steffen A. (2018). Global evaluation and calibration of a passive air sampler for gaseous mercury. Atmos. Chem. Phys..

[6] Kalina J., Scheringer M., Borůvková J. (2017). Passive air samplers As a tool for assessing long-term trends in atmospheric concentrations of semivolatile organic compounds. Environ. Sci. Technol..

[7] Tuduri L., Millet M., Briand O. (2012). Passive air sampling of semi-volatile organic compounds. TrAC Trends Anal. Chem..

[8] Eng A., Harner T., Pozo K. (2014). A prototype passive air sampler for measuring dry deposition of polycyclic aromatic hydrocarbons. Environ. Sci. Technol. Lett..

[9] Markovic M.Z., Prokop S., Staebler R.M. (2015). Evaluation of the particle infiltration efficiency of three passive samplers and the PS-1 active air sampler. Atmos. Environ..

[10] Guéguen F., Stille P., Dietze V. (2012). Chemical and isotopic properties and origin of coarse airborne particles collected by passive samplers in industrial, urban, and rural environments. Atmos. Environ..

[11] Yamamoto N., Hikono M., Koyama H. (2006). A passive sampler for airborne coarse particles. J. Aerosol Sci..

[12] Haig C.W., Mackay W.G., Walker J.T. (2016). Bioaerosol sampling: Sampling mechanisms, bioefficiency and field studies. J. Hosp. Infect..

[13] Mainelis G. (2020). Bioaerosol sampling: Classical approaches, advances, and perspectives. Aerosol Sci. Technol..

[14] Dong H., Wang X., Xu L. (2023). A flow-through passive sampler for microplastics in air. Environ. Sci. Technol..

[15] Szponar N., McLagan D.S., Kaplan R.J. (2020). Isotopic characterization of atmospheric gaseous elemental mercury by passive air sampling. Environ. Sci. Technol..

[16] Zhang X., Brown T.N., Ansari A. (2013). Effect of wind on the chemical uptake kinetics of a passive air sampler. Environ. Sci. Technol..

[17] Klánová J., Èupr P., Kohoutek J. (2008). Assessing the influence of meteorological parameters on the performance of polyurethane foam-based passive air samplers. Environ. Sci. Technol..

[18] Gong P., Wang X., Liu X. (2017). Field calibration of XAD-based passive air sampler on the Tibetan plateau: Wind influence and configuration improvement. Environ. Sci. Technol..

[19] Klánová J., Harner T. (2013). The challenge of producing reliable results under highly variable conditions and the role of passive air samplers in the Global Monitoring Plan. TrAC Trends Anal. Chem..

[20] Meierdierks J., Zarfl C., Beckingham B. (2021). Unique calibration of passive air sampling for field monitoring of PAHs with polyethylene thin films across seasons and locations. Environ. Sci.: Atmos..

[21] Abdallah M.A.-E., Harrad S. (2010). Modification and calibration of a passive air sampler for monitoring vapor and particulate phase brominated flame retardants in indoor air: Application to car interiors. Environ. Sci. Technol..

[22] Melymuk L., Robson M., Helm P.A. (2011). Evaluation of passive air sampler calibrations: Selection of sampling rates and implications for the measurement of persistent organic pollutants in air. Atmos. Environ..

[23] Fan Z.-H.T. (2011). Passive air sampling: Advantages, limitations, and challenges. Epidemiology.

[24] Wang Z.-M., Zhou Y., Gaspar F.W. (2020). Using low cost open-face passive samplers to sample PM concentration and elemental composition in childcare facilities. Environ. Sci.: Process. Impacts.

[25] Xiao H., Hung H., Harner T. (2007). A flow-through sampler for semivolatile organic compounds in air. Environ. Sci. Technol..

[26] Lin C., Becker S., Timmis R. (2011). A new flow–through directional passive air sampler: Design, performance and laboratory testing for monitoring ambient nitrogen dioxide. Atmos. Pollut. Res..

[27] Xiao H., Hung H., Harner T. (2008). Field testing a flow-through sampler for semivolatile organic compounds in air. Environ. Sci. Technol..

[28] Manibusan S., Mainelis G. (2022). Passive bioaerosol samplers: A complementary tool for bioaerosol research. A review. J. Aerosol Sci..

[29] Drdova S., Zhao S., Giannakou M. (2022). Biomimetic light-driven aerogel passive pump for volatile organic pollutant removal. Adv. Sci..

[30] Zhao S., Siqueira G., Drdova S. (2020). Additive manufacturing of silica aerogels. Nature.

[31] Ghaffari-Mosanenzadeh S., Aghababaei Tafreshi O., Karamikamkar S. (2022). Recent advances in tailoring and improving the properties of polyimide aerogels and their application. Adv. Colloid Interface Sci..

[32] Qian Z., Wang Z., Chen Y. (2018). Superelastic and ultralight polyimide aerogels as thermal insulators and particulate air filters. J. Mater. Chem. A.

[33] Zhai C., Jana S.C. (2017). Tuning porous networks in polyimide aerogels for airborne nanoparticle filtration. ACS Appl. Mater. Interfaces.

[34] Qiao S., Kang S., Zhang H. (2021). Reduced shrinkage and mechanically strong dual-network polyimide aerogel films for effective filtration of particle matter. Sep. Purif. Technol..

[35] Qiao S., Kang S., Zhu J. (2021). Facile strategy to prepare polyimide nanofiber assembled aerogel for effective airborne particles filtration. J. Hazard. Mater..

[36] Wu T., Ganobjak M., Siqueira G. (2023). 3D Printed polyimide nanocomposite aerogels for electromagnetic interference shielding and thermal management. Adv. Mater. Technol..

[37] Meador M.A.B., Alemán C.R., Hanson K. (2015). Polyimide aerogels with amide cross-links: A low cost alternative for mechanically strong polymer aerogels. ACS Appl. Mater. Interfaces.

[38] Kantor Z., Wu T., Zeng Z. (2022). Heterogeneous silica-polyimide aerogel-in-aerogel nanocomposites. Chem. Eng. J..

[39] Peters T.M., Sawvel E.J., Willis R. (2016). Performance of passive samplers analyzed by computer-controlled scanning electron microscopy to measure PM10–2.5. Environ. Sci. Technol..

[40] Gaga E.O., Harner T., Dabek-Zlotorzynska E. (2019). Polyurethane foam (PUF) disk samplers for measuring trace metals in ambient air. Environ. Sci. Technol. Lett..

[41] Zhao Y.-B., Cen T., Tang J. (2022). An elution-based method for estimating efficiencies of aerosol collection devices not affected by their pressure drops. Sep. Purif. Technol..

[42] Bian Y., Wang S., Zhang L. (2020). Influence of fiber diameter, filter thickness, and packing density on PM2.5 removal efficiency of electrospun nanofiber air filters for indoor applications. Build. Environ..

[43] Liu T., Liang F., Chen S. (2023). Aramid reinforced polyimide aerogel composites with high-mechanical strength for thermal insulation material. Polym. Adv. Technol..

[44] Broßell D., Tröller S., Dziurowitz N. (2013). A thermal precipitator for the deposition of airborne nanoparticles onto living cells—Rationale and development. J. Aerosol Sci..

[45] Ratismith W., Ali D., Briggs J.S. (2021). A non-tracking semi-circular trough solar concentrator. Int. J. Energy Res..

[46] Gupta M., Dubey A.K., Kumar V. (2019). Solar concentrator based multipurpose sunlight harvesting system without tracking. OSA contin.

