# Supplementary material for: An aerogel-based light-driven knudsen pump approaching complete passive capturing of airborne particles in wide size ranges
Source: Fundam Res. 2025 Apr 18;6(5):3241–50. doi: 10.1016/j.fmre.2025.04.006 (PMC13644413; doi:10.1016/j.fmre.2025.04.006)
Supplement: Supplementary file 1 [file mmc1.docx]

**Supporting Information for**

**An Aerogel-Based Light-Driven Knudsen Pump Approaching Complete Passive Capturing of Airborne Particles in Wide Size Ranges**

Yi-Bo Zhao^a,b,1^, Tingting Wu^c,d,1^, Céline Heimhofer ^a,b^, Natascha Heppeler ^a,b^, Min Gao^a,b^, Tianyu Cen^e,f^, Fabian Weyand^a,b^, Christian Ludwig^e,f^, Shanyu Zhao^c,*^ and Jing Wang^a,b,*^

a. Institute of Environmental Engineering, ETH Zürich, Zürich 8093, Switzerland

b. Laboratory for Advanced Analytical Technologies, Empa, Swiss Federal Laboratories for Materials Science and Technology, Dübendorf 8600, Switzerland

c. Laboratory for Building Energy Materials and Components, Empa, Swiss Federal Laboratories for Materials Science and Technology, Überlandstrasse 129, Dübendorf 8600, Switzerland

d. Institute of Sustainability for Chemicals, Energy and Environment (ISCE^2^), Agency for Science, Technology and Research (A*STAR), 2 Fusionopolis Way, Innovis #08-03, 138634, Singapore

e. Laboratory for Sustainable Energy Carriers and Processes (LEP), Energy and Environment Research Division (ENE), Paul Scherrer Institut (PSI), Villigen PSI 5232, Switzerland

f. Environmental Engineering Institute (IIE, GR-LUD), School of Architecture, Civil and Environmental Engineering (ENAC), École Polytechnique Fédérale de Lausanne (EPFL), Lausanne 1015, Switzerland

^1^These authors contributed equally to the work.

^*^ Corresponding author

Shanyu Zhao, E-mail: [shanyu.zhao@empa.ch](mailto:shanyu.zhao@empa.ch)

Jing Wang, E-mail: [jing.wang@ifu.baug.ethz.ch](mailto:jing.wang@ifu.baug.ethz.ch)

**Fig. S1.** (a) Setup for determining the particle collection efficiencies of the PI aerogel passive sampler. (b) Setup for measuring light-driven airflow rates. (c) Setup for determining the temperatures of the PI aerogel surface.

**Fig. S2.** Size distribution of the NaCl aerosol in the plastic buffer for sampling performance of the PI aerogel passive samplers using (a) SMPS and (b) APS.

**Fig. S3.** Temperature difference between the top (the optical absorber layer) and bottom layer of the PI aerogel passive sampler sample (a) mesoporous, (b) hierarchical, (c) spherical, and (d) spherical/hierarchical as a function of the lamp-to-chamber distance.

**Fig. S4.** Flow rates of the PI aerogel passive sampler sample (a) mesoporous, (b) hierarchical, (c) spherical, and (d) spherical/hierarchical as a function of the lamp-to-chamber distance.

**Fig. S5.** Collection efficiency of the PI aerogel passive sampler sample (a) mesoporous, (b) hierarchical, (c) spherical, and (d) spherical/hierarchical using the SMPS scanning.

**Fig. S6.** Collection efficiency of the PI aerogel passive sampler sample (a) mesoporous, (b) hierarchical, (c) spherical, and (d) spherical/hierarchical using the APS measurement. The low collection efficiency of 2 – 3 µm in Figure S6a was likely due to low particle numbers detected by APS.

**Fig. S7.** Downstream particle number as a function of lamp-to-chamber distance.

**Fig. S8.** Cross-sectional SEM image of (a) original hierarchical sample and EDS mapping of (b) C and (c) O; (d) the hierarchical sample after ambient particle collection for one week and EDS mapping of (e) C and (f) O.

**Fig. S9.** Application of the light-driven hierarchical aerogels in the ambient environment for water-soluble metal analysis (a), and (b) light-driven (with some insects on the metal plate due to the artificial lamp) and (c) control aerogel after 24-day sampling, and (d) the active sampling filter after 24 days with a sampling flow rate of 5.2 L/min, and (e) the control filter.

**Fig. S10.** Size distribution of particle mass and concentrations of water-soluble metals in the ambient environment.

**Fig. S11.** The collection efficiency of the hierarchical PI aerogel as a function of time.
